# Age-related differences in the mental health burden among Ukrainians during war and forced migration

**DOI:** 10.3389/fpubh.2026.1947740

**Published:** 2026-10-15

**Authors:** Spitsyna Larysa

**Affiliations:** Department of Developmental Psychology, Faculty of Psychology, Taras Shevchenko National University of Kyiv, Kyiv, Ukraine

**Keywords:** age-related differences, anxiety, depression, forced migration, mental health burden, PTSD, resources

## Abstract

**Introduction:**

Our study focuses on the effects of the stressors of modern conditions of martial law and forced displacement on the state and mental health of Ukrainians adult. The aim of our study is to analyze the state of mental health and wellbeing of Ukrainians during war and forced migration compared, namely to determine the level of the most common mental health problems - depression, anxiety and post-traumatic stress disorder (PTSD), as well as resources for overcoming the consequences of stressful influences.

**Method:**

We developed a conceptual model of the components of mental health and wellbeing, which became the basis for the development of an online questionnaire on the LimeSurvey platform, with using the PTSD Checklist for DSM-5 (PCL-5), the Life Events Checklist for DSM-5 (LEC-5), the Refugee Post-Migration Stress Scale (RPMS), the Brief Symptom Inventory Derogatis (BSI-18), block of open-ended socio-demographic issues, and SPSS Statistics 21.0.

**Results:**

The sample comprised 306 Ukrainian participants. Participants were grouped by age: under 26 years and 26 years or older; and place of residence: 1) “lives in another country;” 2) “lives in Ukraine, moved;” 3) “lives in Ukraine, did not move.” Differences in symptom scores were observed between subgroups, with the smallest subgroup interpreted cautiously. Among Ukrainian people affected by armed conflicts and migrants, the most common mental disorders (CMDs), such as depression, anxiety disorders and post-traumatic stress disorder, constitute a significant proportion of serious illnesses worldwide. The results obtained in our study confirm this trend also among Ukrainians, who are currently experiencing all the difficulties of wartime.

**Discussion:**

The findings provide a descriptive comparison of psychological symptoms and perceived coping resources across different age and residential groups of Ukrainians adult in the condition of war. Resource-oriented to mental health assessment and intervention for war-affected populations is important, contributing to understanding adaptive functioning in the context of protracted armed conflict and may nevertheless inform future research and support planning for war-affected populations.

## 1 Introduction

The war in Ukraine in recent years has triggered many stressors that have a powerful impact on the wellbeing and mental health of Ukrainians, both those who have remained in Ukraine and those who have been forced to migrate in search of safe haven. Unfortunately, living in Ukraine during the war and seeking safe haven and resettlement have a powerful impact not only on the wellbeing of people, but also on their mental health. And this is natural, as prolonged life in stressful conditions can usually have both immediate and long-term consequences. It has been proven that populations affected by war often experience extreme and chronic stress.

Various consequences of the war, such as the death of a loved one, displacement of family, damage to property or other valuable assets, lack of psychological preparedness for a man-made disaster, lack of social support, negative coping skills, and others, can negatively affect the mental health of the Ukrainian population (1). Thus, according to research, war-affected people are at high risk of mental health complications, including post-traumatic stress disorder (PTSD), anxiety and depression (2, 3). A study by Peek (4) found that children, adolescents and women experience various behavioral, psychological and emotional problems and instability after any type of disaster. Emotional instability, stress reactions, anxiety, trauma and other psychological symptoms are often observed after disasters and other traumatic experiences (5). In a systematic review of the mental health of war refugees, Bogic et al. (6) found that psychological disorders such as post-traumatic stress disorder, depression and unspecified anxiety disorders were generally very common among war refugees, even after years of resettlement.

According to the results of the meta-analysis (5) was estimated that the prevalence of mental disorders (depression, anxiety, post-traumatic stress disorder) was 22.1% at any point in time in the conflict-affected populations assessed. They estimated that approximately one in five people in post-conflict settings has depression, anxiety disorder, post- traumatic stress disorder According to a study conducted among Ukrainian refugees in the Czech Republic: 51% of Ukrainian refugees have depressive conditions, but only 14% admit it by self-assessment; 23% of Ukrainian refugees have high and very high levels of anxiety (7).

Adolescents and young adults are considered by the (8, 50, 56) to be at high risk of mental disorders, including depression, anxiety and conduct disorders. Ukrainian youth are, of course, the most affected by the full-scale invasion, as they are directly responding to the ongoing crisis, while the psychological impact of the war on the populations of neighboring countries, especially in Central Europe, cannot be ignored. A study by nine reported high levels of anxiety, anger, perceived threat and compassion for the Ukrainian people in some NATO countries, i.e., Germany, Poland, the United States (US) and the United Kingdom (UK) (9, 55).

There is evidence that psychological stress, especially prolonged stress, leads to significant changes in behavioral risk factors (BRFs). Armed conflicts contribute to the spread of unhealthy behaviors such as excessive alcohol consumption, smoking, poor eating habits, lack of physical activity and sleep disturbances. These behaviors, in turn, contribute to the development of various diseases and the deterioration of public health. It is clear that young people are one of the most vulnerable groups of the population, along with children and adolescents, who are severely limited by the capabilities of the adults responsible for them. Mental health professionals are increasingly aware of the need to better understand and research the mental health needs of the population in order to be able to respond to them adequately.

It is precisely because of the urgency of mental health problems of the population affected by war and forced migration that we have seen numerous attempts over the past two decades to study, describe and structure the peculiarities of mental health manifestations in the current period.

### 1.1 Analysis of recent research and publications

Aspects of mental health and psychosocial support for forced migrants have been studied in many works. First of all, it is important to note that there is epidemiological evidence that the population during war in their country, as well as refugees and asylum seekers during and after displacement, are disproportionately exposed to extreme stressors compared to other population groups, including significant losses and potentially traumatic events such as torture and war (6). Compared to the general population, refugees have been found to experience significantly higher levels of psychological distress, higher levels of social stress in various domains (economic, demographic, social and cultural, environmental events), as well as higher rates of several mental disorders, including post-traumatic stress disorder (PTSD), depression and anxiety (10). Compared to the general population, psychosis is also more common among people who have experienced trauma and displacement (57, 67).

This is supported by research (58), on stress resulting from both adverse or traumatic experiences prior to migration and difficulties following migration (58). There is evidence that post-traumatic stress disorder (PTSD) and other mental disorders affect at least one in three refugees (10, 48, 53). In a meta-analysis, the prevalence of post-traumatic stress disorder (PTSD) among refugees and asylum seekers was 31.46% (11). A recent study using the ICD-11 found that 36.1% of Syrian refugees and asylum seekers reported complex PTSD (59).

A number of studies have investigated the most common mental health disorders, such as anxiety disorders, acute stress recognitive disorders, personality changes, or post-traumatic stress disorder of the population during war or forced migration (60, 61). Much attention is paid to the study of distress, both before displacement and after moving to a new place (country) of residence (6, 7, 10–14, 62).

These findings are consistent with other studies that have found that anxiety and depression These findings are consistent with other studies that have found that anxiety and depression among people are common responses in people from countries experiencing crisis and war (7, 15–17) High levels of depression and anxiety have been reported among people affected by the war in Ukraine (18), particularly if respondents believe that the crisis will last a long time (19). Increased levels of depression and a tendency toward addictive behaviors (such as alcohol and drug abuse) have been reported among Ukrainian university students (17).

In recent years, many authors have naturally focused on the study of mental health problems associated with the full-scale Russian war in Ukraine, which has become one of the largest humanitarian emergencies since World War II, as statistics show that in the first 6 months of the war (2022) alone, more than 6.7 million Ukrainian refugees fled their country (20). The United Nations Children's Fund estimates that 0.5 million children became refugees in the first week of the conflict alone, 1 and the number has been growing rapidly since then.

Armed conflicts are generally perceived as public health emergencies, man-made disasters that require a significant level of international cooperation in the field of health, including mental health (21, 22).

Interestingly, the scientific-based reported prevalence of psychiatric disorders in children, adolescents, and young adults varies considerably. The prevalence of posttraumatic stress disorder (PTSD), anxiety, and depression ranges from 7.6 to 68.9%, 23.7 to 94.9%, and 4.1 to 41.2%, respectively. These significant variations may be explained by culturally mediated factors, methodological issues, and the use of different psychiatric assessment instruments.

Studies have also shown that children from low socioeconomic status families including a large number of children of forced migrants reported higher levels of distress, and that experiencing more of traumatic events increased the risk of developing PTSD. Strong social support, high levels of parental education, and positive coping strategies have been shown to be protective factors that decrease the risk of developing of PTSD and other psychological symptoms (23, 47).

Interesting results regarding the mental health problems of young people in conditions of crisis and stress in Lebanon were obtained in a systematic review of studies on this issue (24). Namely, a connection was found between the manifestations of anxiety, depression and stress, with physical pain, in young people living in crisis conditions of political and economic instability of the country.

According to the statistics data, in the world 10%−20% of people aged 18–29 have mental health problems that are associated with the feeling of physical pain. The article also compares the results of studies on this problem in Lebanon with similar studies in WEIRD countries. The data show a two-way relationship between mental health and physical pain, and that university students suffer the most from this (36.1% of studies), and 81% of studies reported a higher prevalence of pain among women. As already noted, prolonged psychological stress, one type of which is post-migration stress, leads to hormonal dysregulation, including elevated cortisol levels, which can disrupt cardiovascular and metabolic processes (25, 26), and also increase unhealthy behaviors and bad habits.

These trends are reinforced by behavioral risk factors among Ukrainians during the war, which are exacerbated by existing stressors. According to the study results, military conflicts and the stress associated with them often lead to lifestyle changes and an increase in behavioral risk factors, such as eating behavior problems, tobacco and alcohol abuse, gambling addiction, etc., problems that are also relevant for adolescents and young people (27).

In particular, according to the study, 34.7% of the subjects who constantly experienced the stress of being in war conditions reported eating problems, while 22.4% experienced weight gain and 36.2% experienced weight loss. In addition, 13.3% and 16.8% reported an increase in smoking and alcohol consumption, respectively. Sleep disturbances were reported by 72.4% of respondents, while 55.6% reported a decrease in physical activity. These are behavioral risk factors that are directly related to a decrease in quality of life of being in war conditions, social maladjustment, and traumatic experiences.

Acculturation stress, caused by separation from family due to forced migration, disruption of social ties, social isolation, changing gender roles, unemployment, loss of social status (28, 29), as well as the need to learn to navigate in a completely new community and cultural system (63), is also often the subject of research in clinical psychology in recent years. Much research is devoted to the specifics of culturally mediated mental health problems of forced migrants and refugees. The problems of mental health and psychosocial wellbeing of Syrian refugees were studied by Sijbrandij et al. (64), Eva et al. (30), the problems of Iranian refugees in the field of mental health were studied by Arnetz et al. (65), refugees - speakers of Arabic and Farsi, who are in Germany - Boettcher and Neuner (31), the problems of Ukrainian forced migrants - Fritschi et al. (32), BFS Bundesamt (68), Kovalenko et al. (33), Spitsyna (34), Długosz (35) and others.

The results of the Polish study 2022 show that depression, anxiety disorders and post-traumatic stress disorder can be observed in 73% of respondents of Ukrainian forced migrants, 66% of respondents demonstrate psychological distress, with young respondents experiencing higher psychological distress (35). Despite the relevance and large number of studies on mental health problems in stressful and emergency situations, there has been practically no research on the most common mental health problems (CMDs) – depression, anxiety, post-traumatic stress disorder among Ukrainians, during war and forced migration.

Therefore, the purpose of our study was to describe symptoms of the most common mental health problems - depression, anxiety, post-traumatic stress disorder and subjectively perceived stress-coping resources among Ukrainians during war and forced migration, with comparisons by age group and place of residence.

## 2 Methods

For the purposes of our study, we developed a conceptual model of mental health and wellbeing components, which became the basis for the development of an online questionnaire using the LimeSurvey platform, consisting of a series of standardized questionnaires and several blocks of open-ended socio-demographic issues, which we will name below.

The questionnaire included the PTSD Checklist for DSM-5 (PCL-5), which captures symptoms of Post Traumatic Stress Disorder and the Life Events Checklist for DSM-5 (LEC-5) (36), which is used in our study to identify the list of life events that may have been triggers in the pre-migration stressful situations experienced by our respondents. The Refugee Post-Migration Stress Scale (RPMS) (37) is used for a similar purpose, only to identify post-migration factors and as the indicator of unintended consequences of their stress. Using the Brief Symptom Inventory Derogatis (BSI-18), which is designed to assess symptoms of anxiety, depression and somatisation of our respondents (38), all questionnaires with the Ukrainian versions were validated. Statistical data processing has been carried out using computer software IBM SPSS Statistics for Windows, Version 21.0 (IBM Corp., Armonk, NY, USA).

## 3 Results

The sample included Ukrainians adults, including young adults who were living either in Ukraine or were forced to migrate abroad. Participants were recruited through online channels, including personal and professional networks, social media, and non-governmental organizations, using a non-random sampling strategy (snowball method). Eligible participants were individuals who: (1) were Ukrainian citizens; (2) met the specified age criteria; (3) lived in Ukraine or were abroad following forced displacement/migration; and (4) were able to read the study information and provide electronic informed consent. In addition, incomplete questionnaires were excluded from the calculation of the overall results. Participation was voluntary and anonymous.

A total 306 participants completed the survey (see Table 1). For analysis, participants were divided into two age groups:

**Table 1:** Characteristics of the sample by age and place of residence.

Variable	Category	*N*
Age	age < 26	94
	age ≥ 25	212
Place of residence	Lives in another country	74
	Lives in Ukraine, moved	44
	Lives in Ukraine, not moved	188
	Total	306

1) “age < 26;” 2) “age ≥ 25.”

And also into three groups by place of residence:

1) “lives in another country;” 2) “lives in Ukraine, and has moved to a safer location;” 3) “lives in Ukraine, has not moved.”

In addition, it can be noted that our sample consisted mainly of women, with Ukrainian citizenship, from 18 to 60 years old, the total number of *N* = 290, among which: 1) “lives in another country”- 75 persons; 2) “lives in Ukraine, and has moved to a safer location” - 42; 3) “lives in Ukraine, has not moved” - 173 persons. Besides, the age characteristics of the groups of women we identified are also interesting, namely: the average age of women Age M (SD) who 1) migrated to other countries - 35.3 years; 2) “lives in Ukraine, and has moved to a safer location” - 27.6 years; 3) “lives in Ukraine, has not moved” - 35.0 years.

Also, useful and interesting may be the data on education in our sample, which demonstrated a fairly high level of education among the groups of women we identified, namely: those with higher education, at least a Bachelor's degree, among those who 1) migrated to other countries - 109 people (63.0%); 2) “live in Ukraine and moved to a safer place” - 20 (46.6%); 3) “live in Ukraine, did not move” - 53 (70.7%). These data allow to more fully characterize our study sample, both as a whole and by various subgroups.

Descriptive comparisons showed symptoms of depression, anxiety, and PTSD among our participants. As we have already noted above, among people affected by armed conflicts and forced migrants, the most common mental disorders (CMDs), such as depression, anxiety disorders, and post-traumatic stress disorder, constitute a significant proportion of serious illnesses worldwide (7, 10, 69). The results obtained in our study confirm this trend also among Ukrainians, who are currently experiencing all the hardships of wartime.

The descriptive results indicated differences between subgroups. Results obtained show that Ukrainian forced migrants living in another country have a higher score on the depression scale compared to other population groups (mean 1, 41). Including the group of young migrants under 26 years of age (18–26) (mean 1, 31). This can be seen even more clearly in the diagram Figure 1, compared to the indicators of the groups of people who remained in Ukraine - both those who moved to a safer place and those who stayed at home (Figure 2).

**Figure 1:**
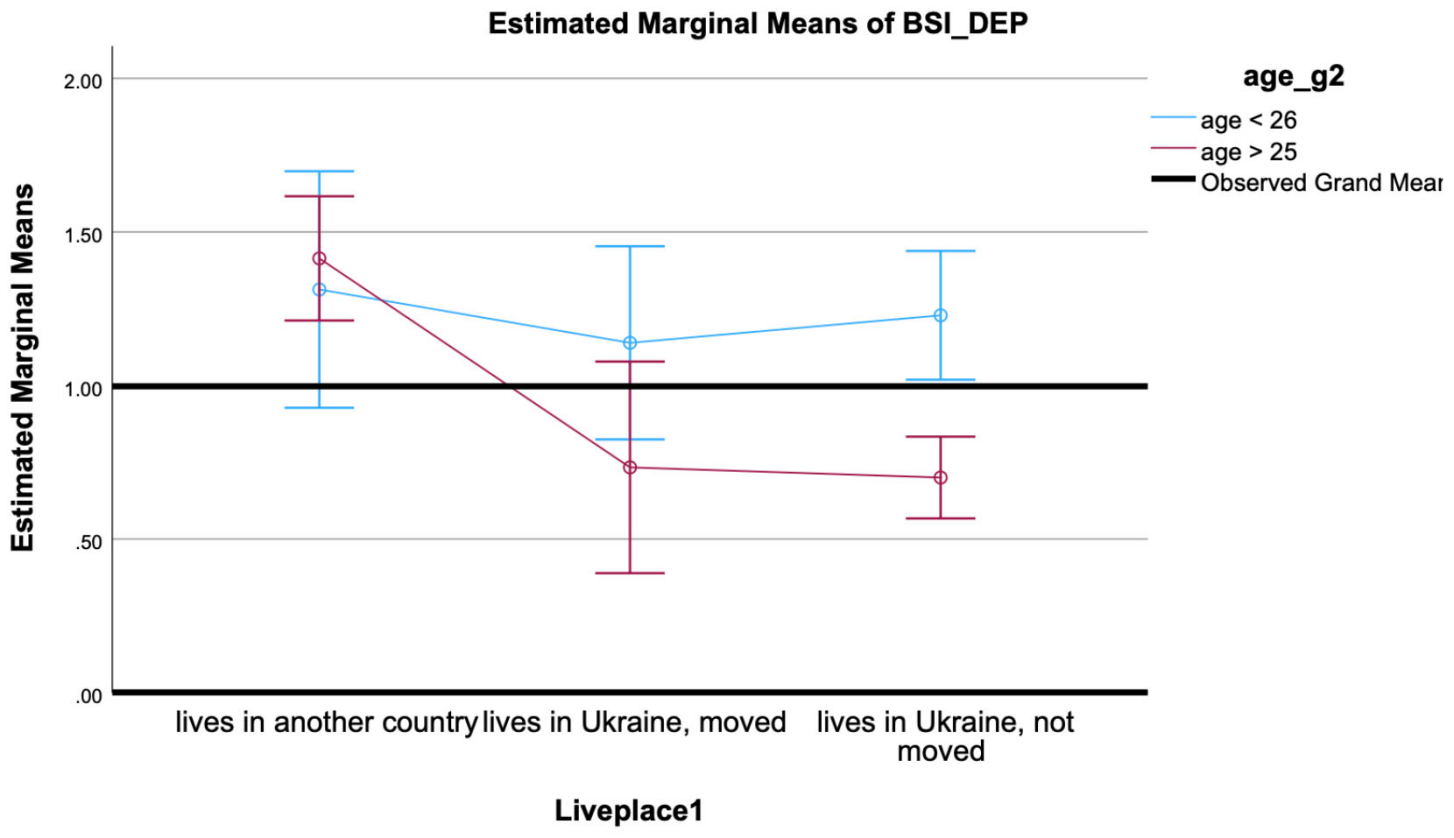
Estimated Marginal Means on depression scale (BSI). level of credibility: *p* ≤ 0.05.

**Figure 2:**
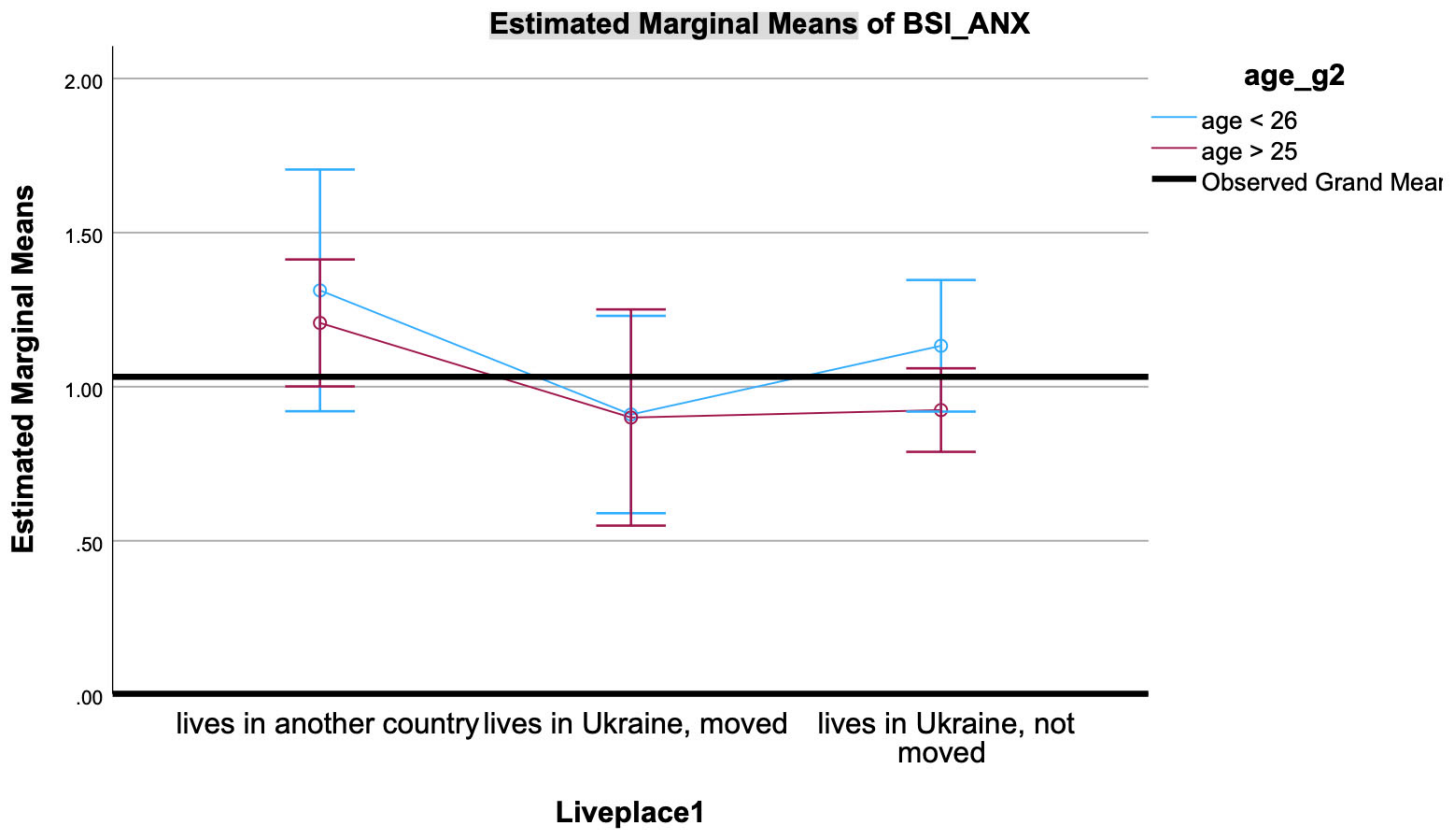
Estimated Marginal Means on anxiety scale (BSI). level of credibility: *p* ≤ 0.05.

The prevalence of depressive/anxiety symptoms among forced migrants is usually higher than among the most at-risk groups in the local population. For example, 39 note in Table 2 their study that among the refugee population, 45% of adults in the Czech Republic have symptoms of moderate depression or moderate anxiety. The mental state of forced migrants is also influenced by the circumstances that preceded their migration (pre-migration factors), whether some of their relatives remained in Ukraine, whether any of the family members suffered: whether all family members are alive, whether they ended up in the zone of direct military action or occupation, whether their property and housing were preserved, whether their work/study was preserved, etc (see Table 3).

**Table 2:** Descriptive statistics on the depression scale for different age groups & place of residence.

Age group	Place of residence	*M*	SD	*n*
Age < 26	Lives in another country	1, 31	0, 93	16
	Lives in Ukraine, moved	1, 14	0, 82	24
	Lives in Ukraine, not moved	1, 23	0, 76	54
	Total	1, 22	0, 80	94
Age ≥ 25	Lives in another country	1, 41	1, 15	58
	Lives in Ukraine, moved	0, 73	0, 59	20
	Lives in Ukraine, not moved	0, 70	0, 57	134
	Total	0, 90	0, 83	212
Total	Lives in another country	1, 39	1, 10	74
	Lives in Ukraine, moved	0, 95	0, 75	44
	Lives in Ukraine, not moved	0, 85	0, 67	188
	Total	1, 00	0, 83	306

M, mean; SD, standard deviation; *n* = sample size. Calculated using alpha = 0.05.

**Table 3:** Descriptive statistics by the anxiety scale for different age groups &place of residence.

Age group	Place of residence	*M*	SD	*n*
Age < 26	Lives in another country	1, 31	0, 88	16
	Lives in Ukraine, moved	0, 91	0, 83	24
	Lives in Ukraine, not moved	1, 13	0, 73	54
	Total	1, 11	0, 79	94
Age ≥ 25	Lives in another country	1, 21	0, 97	58
	Lives in Ukraine, moved	0, 90	0, 67	20
	Lives in Ukraine, not moved	0, 92	0, 74	134
	Total	1, 00	0, 81	212
Total	Lives in another country	1, 23	0, 94	74
	Lives in Ukraine, moved	0, 91	0, 75	44
	Lives in Ukraine, not moved	0, 98	0, 74	188
	Total	1, 03	0, 80	306

*M*, mean; SD, standard deviation; *n* = sample size. Calculated using alpha = 0.05.

We studied these data using the Refugee Post-Migration Stress Scale (RPSM) and a block of open-ended questions from the questionnaire, the results of the analysis of which will be available in subsequent publications.

Symptoms of depression and anxiety are closely related in populations that experience systemic chronic and catastrophic stressors such as war or man-made disasters, as well as in forced migrants (Czech Republic study). Only a minimal number of people have anxiety symptoms without suffering from depressive symptoms. Therefore, the results we obtained with the BSI anxiety scale are very similar to the results with the depression scale.

As we can see from the table, the indicators of the level of anxiety in the youth sample of forced migrants are higher than in all other groups of our Ukrainian respondents - 1.31, compared to 0.9 in the group of young Ukrainians who remained in Ukraine and moved to a safer place. At the same time, it should be noted that anxiety scores were also high in all age groups of Ukrainians who migrated abroad (mean = 1.23).

Overall, according to the results obtained, in the present sample, most participants did not meet the study threshold for elevated PTSD symptoms, whereas approximately 30% showed substantially elevated symptom scores according to the study estimates. These screening results should not be interpreted as clinical diagnoses. Interestingly that young adult Ukrainians demonstrate a significantly lower level of PTSD symptoms than older Ukrainians (see Figure 3). It is in such a situation that preventive and supportive measures in communities can have a good effect in reducing the impact of numerous stressors on young Ukrainians.

**Figure 3:**
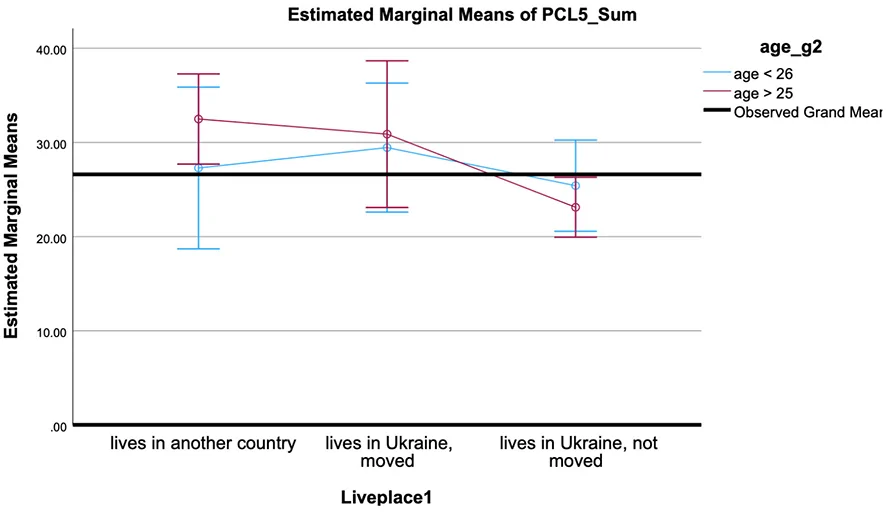
Estimated Marginal Means on PTSD scale (BSI).

It will also be useful to consider the following block of information received regarding resources for overcoming difficult life situations that participants reported using or considering useful for coping with difficult life situations.

In addition to symptoms of mental illness, we also investigated the extent to which young adults, both those who remained in Ukraine and forced migrants, are aware of their available resources to overcome the impact of stressors. A separate block of our online questionnaire with LimeSurvey was dedicated to this aspect. Living under the influence of chronic stressors puts increased demands on young people's resilience and available resources. Therefore, an important issue that we wanted to clarify in our study is the question about resources that respondents prefer to use to overcome difficult life situations. To study the preferred available resources, we suggested the following answer options:

1. Your personal qualities and abilities (1)
2. Your beliefs and convictions (1)
3. Your communication and relationships with others
4. Your work and professional activities
5. Sports and physical activity
6. Cultural activities, creativity, art
7. Support from family members, friends and others (1)
8. Support from mental health professionals
9. Something else (what exactly?)

The full picture of the priorities in the resources used can be seen in Figure 4. We received interesting results about the priorities of our young respondents: in the first place, three resources were preferred, namely: reliance on your beliefs and convictions, personal qualities & abilities and social support - from family members, friends and others. The second place was shared by resources communication and relationships with others and professional activities (job).

**Figure 4:**
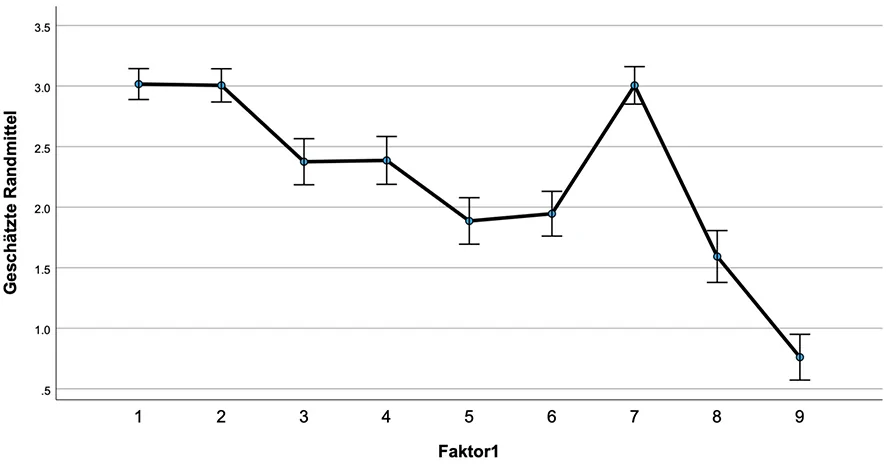
Priorities in available couping resources.

## 4 Discussions

The findings provide a descriptive comparison of psychological symptoms of the most common mental health problems - depression, anxiety, and PTSD and subjectively perceived stress-coping resources among Ukrainians during war and forced migration, with comparisons by age group and place of residence. Resource-oriented to mental health assessment and intervention for war-affected populations is important, contributing to understanding adaptive functioning in the context of protracted armed conflict.

This quantitative cross-sectional study provides a description of depression, anxiety, and PTSD symptoms among Ukrainian participants living in different residence contexts. Differences in levels of symptom manifestation were observed across age and residence subgroups, including a descriptive pattern of higher depression and anxiety scores among participants living abroad.

In whole, our study shows a deterioration in mental health among young Ukrainians, both forced migrants and those who stayed in Ukraine, in terms of symptoms of depression, anxiety and post-traumatic stress disorder (PTSD), which is consistent with the findings of many studies. For example, this is also consistent with the results of a Czech study (39), which found that 23% of refugees suffer from moderate anxiety symptoms, compared to 7% of Czechs. Again, anxiety is more prevalent among women, who make up over 70% of refugees in the Czech Republic. Symptoms of anxiety measured by the GAD-7 questionnaire used in their study include, among others, nervousness, worry, restlessness or irritability.

According to the accumulating evidence of worse mental health in consequence of russian war in Ukraine 2022 (40, 41, 66), Ukrainians unequivocally reported worse levels of all mental health indices compared to bordering and distal countries. Although the rate of Ukrainians screened positive for CPTSD has largely ranged across studies (40–42), in this study, it was higher for Ukrainians and bordering countries than the distal countries. Ukrainians also had the lowest resilience levels, though at comparable levels to similar studies (17).

Importantly, recent peer-reviewed studies has confirmed the association of low resilience with high levels of mental health symptoms in a variety of traumatic/stressful contexts (43, 51, 54). There are also other explanations, such as the cumulative and/or overlapping impact of other factors on mental health (e.g. COVID-19) (66). According to the Conservation of Resources (COR) theory (49), the consequences of military events are intertwined with resource losses, which again and again activate stress reactions (49, 52). That is why, in the context of extreme resource loss in consequence of the war, when researchers note the insurmountable stress and depletion of Ukrainians' resources that results from this (44, 45), we believe it is very important to study the “treasure trove” of conscious resources available for use by Ukrainians right now.

In addition to studying the general level of symptoms of depression, anxiety, PTSD, somatization, etc., our conceptual model included the study of personality problems in the social context, including pre- and post-traumatic factors of migrants' psychological trauma. This allows us to take into account not only the short-term effects of stressors, but also the long-term effects of certain events (stimuli) on a person's life when conceptualizing personal outcomes. The results of our study are in line with those of our Swiss and Ukrainian colleagues. Thus, according to the results of a resent studys in 2023 (32, 68), the situation of Ukrainian refugees in Switzerland is characterized by a really high level of exposure to stress factors, which can still be stabilized in a certain sense with the help of primary support services. It is important to understand that for such a large and diverse group of people as refugees and migrants, there is an increased risk of mental health problems under certain conditions. Some of the most common stressors for this group of internally displaced people in Ukraine include difficulties finding work, financial difficulties, lack of a sense of belonging to the host community, family conflicts, low mood, alcohol abuse, fear of the future, loneliness and feelings of loss - all of which can, and often do, increase the risk of mental health problems. A recent study shows that mental disorders among internally displaced persons are significantly associated with cumulative traumatic experiences, more recent displacement, and poor economic status (46).

Our results indicate deterioration in the mental health of Ukrainians, which is consistent with other studies. In addressing the psychological health of youth, it is important to focus on early systematic identification of problems. The advantage is that, unlike interventions for adults, interventions can be carried out in an educational environment, for example, at a university or college. Interventions should be aimed primarily at ensuring the social conditions of young people's lives and integration into the community, or, if necessary, directing them to professional help.

## 5 Limitations and future research

This study has several limitations. First, sample characteristics varied significantly across subgroups, regarding both place of residence and age, - due to the chosen data collection method. It is evident that the relatively small size and unequal numbers of certain subgroups may, unfortunately, reduce the statistical power of comparisons and make the results more sensitive to potential deviations from the assumptions underlying parametric methods. Consequently, results pertaining to the smallest subgroups should be regarded primarily as preliminary descriptive trends that require confirmation in larger and more balanced samples. This applies particularly to the subgroup of participants under the age of 26 residing abroad (comprising only 16 individuals), consequently, the data obtained for this group can be interpreted only as preliminary. Second, the study relied on self-report scales rather than clinical diagnoses, which is acceptable for psychological and psychosocial research, but also has interpretative limitations in the psychiatric field. Third, it should also be noted that the cross-sectional study design limits the assessment of changes in mental health over time and does not support causal relationships. In addition, access to some sociodemographic and migration variables collected via LimeSurvey was technically limited at the time of analysis; therefore, their potential impact could not be fully explored. These limitations should be taken into account when interpreting the study results.

We hope that most of these limitations can be overcome in future studies, which would benefit from the following. First, it is advisable to use a longitudinal study design to assess changes in mental health over time and distinguish between the effects of war, displacement, and adaptation. Second, future work could include more detailed studies of the effects of socio-economic status, prior mental health status, access to services, etc. Third, it would be desirable to combine online and offline recruitment and implement procedures to check for uniqueness of responses. Finally, resource orientation in mental health assessment and intervention for war-affected people should become an important basis for future research and planning of psychosocial support for Ukrainians.

## 6 Conclusions

The findings provide a descriptive comparison of psychological symptoms and perceived coping resources across different age and residential groups of Ukrainians adult in the condition of war. Our study showed that Ukrainians, both those who forced migrated abroad and those who have stayed in Ukraine, have higher rates of manifestation of the most common mental health problems (CMDs), including depression, anxiety disorders and post-traumatic stress disorder. These scores are higher for young Ukrainians who have migrated on depression and anxiety scales. It is also important that, given the context of protracted armed conflict, loss and depletion, resource-oriented in mental health assessment and intervention for war-affected people is important, contributing to understanding adaptive functioning in these conditions and may be a good basis for future research and planning psychosocial support for Ukrainians.

## Data Availability

The raw data supporting the conclusions of this article will be made available by the authors, without undue reservation.
